# Clinical and treatment patterns of advanced and radioiodine-refractory differentiated thyroid cancer. ERUDIT study

**DOI:** 10.1007/s12094-025-04122-6

**Published:** 2025-11-19

**Authors:** Carlos López López, Marcel Sambo, Lorenzo Orcajo-Rincon, Manuel Durán-Poveda, Julio Rodríguez-Villanueva García, Rita Joana Santos, Marta Llanos Muñoz, Elena Navarro-González, Javier Aller, Virginia Pubul, Sonsoles Guadalix, Guillermo Crespo, Cintia González, Carles Zafón, Miguel Navarro, Javier Santamaría Sandi, Ángel Segura, Pablo Gajate, Marcelino Gómez-Balaguer, Javier Valdivia, Jordi L. Reverter, Juan Carlos Galofré, Beatriz Castelo, María José Villanueva, Iñaki Argüelles, Juan Antonio Vallejo Casas

**Affiliations:** 1 Department of Medical Oncology, Marqués de Valdecilla University Hospital, IDIVAL, UNICAN, Avda Valdecilla 28, 39008 Santander, Cantabria Spain; 2 Department of Endocrinology, Gregorio Marañón University Hospital, Madrid, Spain; 3 DHBL Oncology, Clinical Evidence Generation – Eisai Farmacéutica S.A, Madrid, Spain; 4 Department of General and Digestive Surgery, Rey Juan Carlos University Hospital, Madrid, Spain; 5 Department is Endocrinology, Hospital CUF Cascais, Cascais, Portugal; 6 Department of Medical Oncology, Hospital Universitario de Canarias, La Laguna, Santa Cruz de Tenerife, Spain; 7 Department of Endocrinology, Virgen del Rocío University Hospital, Seville, Spain; 8 Department of Endocrinology, Puerta de Hierro-Majadahonda University Hospital, Madrid, Spain; 9 Department of Nuclear Medicine, University Hospital and Health Research Institute of Santiago de Compostela, Santiago de Compostela, Spain; 10 Department of Endocrinology and Nutrition, Hospital Universitario, 12 de Octubre, Madrid, Spain; 11 Department of Medical Oncology, Burgos University Hospital, Burgos, Spain; 12 Department of Endocrinology and Nutrition, Consortium of the General University Hospital, CIBER-BBN, Valencia, Spain; 13 Department of Endocrinology and Nutrition, Vall d’Hebron University Hospital and Autonomous University of Barcelona (UAB), Barcelona, Spain; 14 Department of Medical Oncology, University Hospital of Salamanca, Salamanca, Spain; 15 Department of Endocrinology, Cruces University Hospital, Vizcaya, Spain; 16 Department of Medical Oncology Unit, La Fe University and Politecnic Hospital, Valencia, Spain; 17 Department of Medical Oncology, Ramon y Cajal University Hospital, Madrid, Spain; 18 Department of Endocrinology, Doctor Peset University Hospital, Valencia, Spain; 19 Department of Oncology, University Hospital Centre Virgen de Las Nieves, Granada, Spain; 20 Endocrine and Nutrition Service, Health Sciences Research Institute and University Hospital Germans Trias I Pujol, Badalona, Spain; 21 Department of Endocrinology, Clínica Universidad de Navarra, University of Navarra, Pamplona, Spain; 22 Department of Medical Oncology, La Paz University Hospital, Madrid, Spain; 23 Department of Medical Oncology, Alvaro Cunqueiro University Hospital Complex, University of Vigo, Vigo, Spain; 24 Department of Endocrinology and Nutrition, Son Espases University Hospital, Palma, Spain; 25 Department of Nuclear Medicine (UGC), Maimónides Institute of Biomedical Research of Córdoba (IMIBIC), Reina Sofía University Hospital, University of Córdoba, Córdoba, Spain

**Keywords:** Radioiodine-refractory differentiated thyroid cancer, Advanced differentiated thyroid cancer, Epidemiological study, Survival prognostic factors, ERUDIT study

## Abstract

**Background:**

Radioiodine-refractory (RR) disease drives morbidity and mortality in advanced differentiated thyroid cancer (aDTC) and yet real-world longitudinal data from the Iberian Peninsula are sparse.

**Objectives:**

To characterize clinical features, multidisciplinary management, and outcomes of RR-DTC within a multicenter Iberian cohort, and to explore prognostic factors, including the impact of de novo versus recurrent/progressive presentation.

**Methods:**

Multicenter, retrospective cohort of adults with aDTC diagnosed in 2007–2012 at 23 Spanish/Portuguese centers, with follow-up until 2017. Baseline demographic and clinical characteristics, local/systemic treatments used, progression-free survival (PFS) on first-line systemic therapy (ST), overall survival (OS) from RR-DTC, and survival prognostic factors were assessed. An exploratory illness–death multistate Markov model complemented Kaplan–Meier analyses for mortality from initial diagnosis. Missing data were not imputed.

**Results:**

Of 213 aDTC patients, 165 (77.5%) met ≥ 1 RR-DTC criterion at any time during their follow-up. At RR-DTC diagnosis, ECOG was recorded in 92/165 (55.8%): 75 (81.5%) ECOG 0–1 and 17 (18.5%) ECOG 2–4. Management included watchful waiting (WW) in 79/165 (47.9%), locoregional therapy in 83/165 (50.3%), and ST in 61/165 (37.0%), predominantly sorafenib. Median PFS on first-line ST was 16.0 months (95% CI 9.2–29.9). Median OS from RR-DTC diagnosis was 4.7 years (3.4–8.0), shorter for de novo aDTC versus recurrent/progressive eDTC (3.0 vs 8.0 years; log-rank p < 0.0001). In multivariable analyses, age < 55 years and WW ≥ 30 months were associated with longer OS, while de novo aDTC and ECOG 2–4 were associated with higher mortality. From initial DTC diagnosis, the multistate model showed higher cumulative mortality for RR-DTC versus non-RR-DTC (HR 3.22; *p* < 0.001).

**Conclusions:**

In this Iberian cohort, RR-DTC was frequent and often managed within MDTs, with substantial WW use. De novo aDTC and poor performance status were associated with worse survival. Prolonged WW appeared associated with longer OS, but this likely reflects selection and lead/immortal-time biases underscoring the need for prospective validation.

**Supplementary Information:**

The online version contains supplementary material available at 10.1007/s12094-025-04122-6.

## Introduction

Around 90% of diagnosed differentiated thyroid cancers (DTC) have favourable outcomes after surgery and I-131 radioiodine (RAI) based treatments, but 5–10% of them will become locally advanced and/or metastatic [1–3]. One third of the metastatic tumours will not show response to RAI at diagnosis, and nearly two thirds of the remaining will become refractory to this treatment modality (radioiodine-refractory differentiated thyroid cancer; RR-DTC) along follow up [1, 4].

Advanced (locally unresectable or metastatic) RR-DTC population has clearly worse prognosis, with a 10-year survival rate dropping from 56 to 10% after RR diagnosis, median survival significantly reducing to a range of 3 to 9 years depending on the series reviewed, and with a markedly poorer quality of life. [5, 6] Therapeutic options are limited for these patients and all of them have a purely palliative intention [7]. In this sense, the approved multi-tyrosine kinase inhibitors (MKIs), lenvatinib, sorafenib and, more recently cabozantinib, have demonstrated to significantly benefit RR-DTC patients improving progression-free survival (PFS) in early and late stages of advanced refractory disease [8–10]. However, the rate of adverse events associated with these therapies is relatively high [11]. In this context, guidelines for the management of RR-DTC emphasise the role of multidisciplinary teams (MDTs) in deciding the right time to start treatment with MKIs after properly evaluating benefits against potential toxicities [3, 12, 13]. Although treatment decisions within MDTs have become common practice in Spain [13, 14] there are yet no published registries recounting this experience in newly diagnosed advanced DTC (aDTC) and RAI-refractory disease specifically. Similarly, not much information on the natural evolution of this disease and its prognostic factors is yet available from other European sources [13].

Aiming to feel this gap, the ERUDIT study was designed to characterise the natural evolution of adult patients diagnosed with aDTC in Spain and Portugal. Primary results of ERUDIT identified early poor treatment-dependent prognostic factors impacting both the pace at which early disease progresses into advanced stages and the overall survival of aDTC once diagnosed [15]. This suggests, that intensified follow-up practices and therapeutic policies might reverse this trend and should be considered in poor-performing patients [10].

In this communication, we describe the demographic and clinical characteristics, percent use and efficacy profile of therapies used to treat aDTC patients who became RR-DTC. We will also explore potential prognostic factors associated to the appearance of RAI refractoriness, PFS (PFS) from RR, and overall survival (OS) in this population.

## Methods

### Study design and setting

**Fig. 1:**
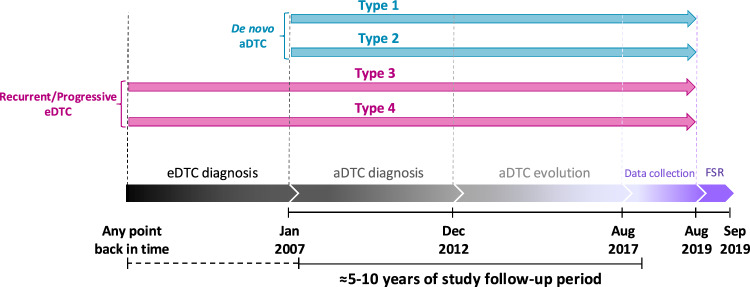
ERUDIT study timeline and classification of aDTC patients according to the type of their diagnosis. *Type 1*: de novo metastatic with resectable locoregional disease. *Type 2*: de novo locoregional unresectable disease. *Type 3*: recurrent metastatic without resectable locoregional disease. *Type 4*: recurrent locoregional unresectable disease, with or without distant metastases. *aDTC* advanced differentiated thyroid cancer, *eDTC* early-stage differentiated thyroid cancer, *FSR* final study report

ERUDIT is a retrospective, international, observational, and longitudinal study involving twenty-three representative hospitals from Spain and Portugal. Clinical records from eligible patients diagnosed with aDTC from January 2007 to December 2012, both inclusive, were retrospectively reviewed until August 2017 (expected 5 to 10 years follow-up) and collected from August 2017 to August 2019 (Fig. 1).

### Patients

Patient records were considered eligible for the present study if affected with aDTC [papillary, follicular, Hürthle’s cell (currently named oncocytic carcinoma), [16] mixed or poorly differentiated] diagnosis was established with first evidence of unresectable locally advanced and/or metastatic disease [presenting either de novo at initial DTC diagnosis or relapsed/progressed after first treatment(s)] was as such documented during the inclusion period. Accordingly, protocol (EIS-CDT-2017–01) eligibility criteria for the study population covered most common aDTC scenarios: *Type 1*, de novo metastatic with resectable locoregional disease; *Type 2*, de novo locoregional unresectable disease; *Type 3*, recurrent metastatic with resectable locoregional disease; and *Type 4*, recurrent locoregional unresectable disease, with/out distant metastases [15]. Informed consent was obtained from patients alive and under active follow-up at the time of data collection. The study was performed in compliance with all basic principles of the Helsinki Declaration (2013) (Accessed November 4, 2023; https://www.wma.net/policies-post/wma-declaration-of-helsinki-ethical-principles-for-medical-research-involving-human-subjects/)[17]. Applicable national approvals were obtained from the accredited Research Ethics Committee of Hospital Universitario Gregorio Marañón (Madrid, Spain) and the National Ethics Committee for Clinical Research (Portugal) prior to the initiation of data collection.

The RR-DTC cohort was comprised of patients who met at least one of the most commonly accepted clinical criteria for refractoriness to radioactive iodine occurring at any time during the course of disease within the identification window of this study. Thus, RR-DTC was defined using the widely accepted consensus criteria from the American Thyroid Association, the European Association of Nuclear Medicine, the Society of Nuclear Medicine and Molecular Imaging, and the European Thyroid Association [18] as follows: (1) no RAI uptake in locoregional recurrence and/or distant metastases at initial detection; (2) progressive loss of RAI uptake after prior avidity; (3) mixed uptake pattern across metastases; (4) radiological progression within 12 months of adequately dosed RAI despite substantial uptake; (5) significant 18F‑FDG uptake; (6) cumulative administered RAI dose > 600 mCi; (7) unresectable primary DTC; and/or (8) aggressive histology consistent with RR behaviour. Criteria were not mutually exclusive; the distribution by criterion is provided in Table 1.

**Table 1:** Demographic and clinical characteristics of the radioiodine-refractory population (*N* = 165)

Parameter	de novo aDTC (*N* = 85)	Recurrent/progressive eDTC (*N* = 80)	Global RAI-refractoriness DTC Population (*N* = 165)
RR-DTC diagnosis, patients *n* (%)			
At first DTC diagnosis	12 (14.1)	1 (1.2)	13 (7.9%)
At follow-up	73 (85.9)	79 (98.8)	152 (92.1%)
RAI refractoriness criteria ^a^, patients *n* (%)			
No RAI uptake in at least 1 lesion	53 (62.3)	60 (75.0)	113 (68.5)
PD despite substantial RAI uptake ^b^	28 (32.9)	18 (22.5)	46 (27.9)
Cumulative RAI dose ≥ 600 mCi	26 (30.6)	20 (25.0)	46 (27.9)
Others ^c^	29 (34.1)	50 (62.5)	79 (47.9)
ECOG status recorded ^d^, patients *n* (%)	52 (61.2)	40 (50.0)	92 (55.8)
ECOG 0–1	37 (71.2)	38 (95.0)	75 (81.5)
ECOG 2–4	15 (16.3)	2 (5.0)	17 (18.5)
Usage of imaging diagnostic methods, ^e,f^ patients *n* (%)			
At least method	38 (44.7)	43 (53.8)	81 (49.1)
CT	21 (55.3)	12 (27.9)	33 (40.7)
^18^FDG-PET	6 (15.8)	16 (37.2)	22 (27.2)
WBS	7 (18.4)	11 (25.6)	18 (22.2)
Others	4 (4.7)	4 (5.0)	8 (4.8)

^a^RAI refractoriness criteria [18] are not mutually exclusive allowing for a patient to be counted in more than one; ^b^ progressive disease observed within 1 year of RAI treatment; ^c^this category includes significant uptake of 18F-fluoro-2-deoxy-D-glucose positron emission tomography, unresectable primary tumour, aggressive DTC histologies such as non-differentiated, Hürthle cell carcinoma (currently named oncocytic carcinoma), [16] or insular carcinomas; ^d^ data refer to patients with RR-DTC confirmation either at first DTC diagnosis or follow-up with ECOG status recorded; ^e^ criteria are not mutually exclusive allowing for a patient to be counted in more than one; ^f^ data refer to patients with RR-DTC confirmation either at DTC diagnosis or follow-up, not to total patient group type N

*aDTC* advanced differentiated thyroid cancer, *CT* computed tomography, *eDTC *early-stage of differentiated thyroid cancer, *ECOG* eastern cooperative oncology group, *PD* progressive disease, ^18^*FDG-PET* ^18^F-fluoro-2-deoxy-D-glucose positron emission tomography, *RAI* radioactive iodine (I-131), *RR-DTC*, radioiodine-refractory differentiated thyroid cancer, *WBS* whole body scan

## Variables at radioiodine-refractoriness disease and clinical endpoints

*Demographic and clinical characteristics*: initial aDTC diagnosis type (de novo or progressive/recurrent), RR-DTC diagnosis (at first DTC diagnosis or at follow-up), RAI refractoriness criteria, [18] eastern cooperative oncology group (ECOG) status, [19] and imaging methods used for diagnosis.

*Therapeutic approach for advanced/RAI-refractory disease and response*: a) Local treatment, b) watchful waiting (WW), and c) systemic therapy (ST).

*Medical specialities involved in advanced/RR-DTC management:* department responsible for patient care and the existence (or absence) of MDT to aid decision making.

*Clinical endpoints of interest:* time since initial RAI treatment to RR-DTC diagnosis, time from RR-DTC diagnosis to start of ST, PFS defined as the time between the start of ST to date of disease progression or death as documented in patient medical history; and OS of RR-DTC, defined as the time from the diagnosis of RAI refractoriness to death from any cause.

Disease progression was defined by objective radiological progression of measurable disease or unequivocal disease progression of non-measurable disease according to the Response Evaluation Criteria in Solid Tumours (RECIST) v1.1 per physician-reported best response to treatment at the end of any given line of systemic therapy (ST). Start and end dates, as well reason for discontinuation of any given ST: ‘progressive disease’ or ‘death’ as registered in medical records, were used to assess tumour progression and progression-free survival.

Minimally progressive disease was defined as radiological progression of measurable disease not meeting RECIST v1.1 thresholds to be declared as objective disease progression or unequivocal disease progression of non-measurable disease without associated clinical deterioration.

*Potential confounding variables:* a total of 33 potential covariates were analysed using the Kaplan–Meier (K–M) method. Later, all covariates were entered into a multivariable Cox model following both forwards addition and backwards removal of non-significant covariates with a type I error ≤ 1. Potential covariates included variables at initial diagnosis (Supplementary Table 1), [15] variables with expected impact on relapse or progression at the early stage of DTC (eDTC) into aDTC, and those likely associated survival outcomes in the advanced disease/RAI-refractory.

## Statistical methods

Based on the generally accepted assumption that approximately 30% of all aDTC become RAI refractory after diagnosis in the next 10 years, an estimated study sample size close to 300 patients (originally considered representative of nearly 20% of all aDTC diagnoses made in Spain and Portugal), was estimated in order to allow for the collection of nearly 100 cases of RR-DTC during the inclusion (January 2007 to December 2012, both inclusive) and follow-up (until August 2017) periods.

Description of clinical characteristics, treatments received by the RR-DTC cohort, and their time-to-event outcomes were analysed. All data were summarised using adequate descriptive statistics (mean, standard deviation; median, quartiles, 95%-confidence intervals (95%CI); minimum and maximum for continuous variables; and absolute and relative frequencies for categorical variables). Because of the retrospective, multicentre nature of the cohort and the risk of unverifiable assumptions, we did not impute missing covariates or times. For ECOG at RR‑DTC diagnosis, the Cox model included an explicit ‘missing’ category to retain patients and reduce bias from complete‑case analyses. We report the extent of missingness for key variables and discuss potential direction and magnitude of bias in the limitations at discussion section.

PFS and OS functions were analysed and compared using Kaplan–Meier (K-M) and Mantel–Haenszel (log-rank) methods. Beyond Kaplan–Meier and Cox models, we additionally applied an exploratory, unidirectional illness–death multistate Markov model [20] to approximate the cumulative incidence of death from initial DTC diagnosis while explicitly accounting for the intermediate, time-dependent RR-DTC state and the competing pathway of death without RR. This framework estimates transition-specific hazards and Aalen–Johansen state/transition probabilities under right-censoring, thereby reducing immortal-time/lead-time bias inherent to grouping patients by eventual RR status.

Prognostic risk factors for OS from RR-DTC diagnosis were analysed by univariate and multivariable Cox proportional risk-based regression models. For multivariable OS from RR-DTC diagnosis, we specified a parsimonious Cox model with four covariates (age < 55 vs ≥ 55, de novo vs recurrent/progressive, ECOG 2–4 vs 0–1/missing, WW ≥ 30 vs < 30 months). With 79 deaths observed in the RR-DTC cohort, the events-per-variable ratio was ≈19.8. Non-evaluable medians in K–M curves were identified as NE. Data were analysed using SAS Institute Inc. Version 8.2 software (Cary, NC. USA). Two-sided *p* < 0.05 was considered statistically significant.

Data abstraction adhered to a standardized case report form. Data management and statistical analyses were conducted by an independent contract research organization with predefined quality checks. The sponsor had no role in individual-level data access, model selection, or inferential decisions. Interpretation was investigator-driven and finalized by consensus of non-industry authors.

## Results

**Fig. 2:**
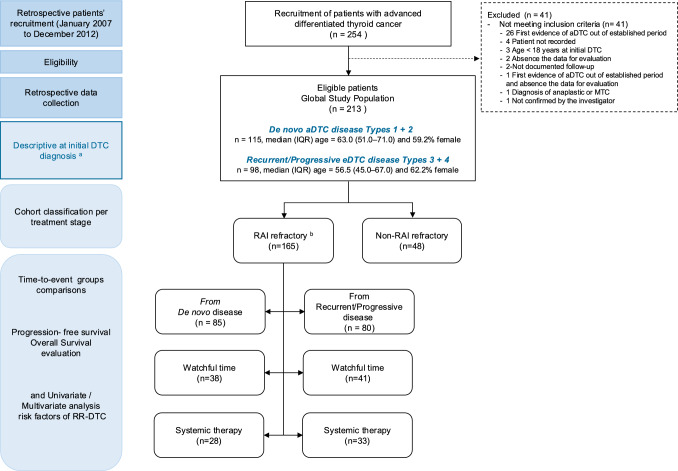
Flow diagram of eligible patients and analysed groups with evaluable data since the diagnosis of advanced differentiated thyroid cancer (aDTC); a, results published at Vallejo et al., 2022 [15]; b, *RAI* refractoriness criteria [18], *eDTC* early-stage differentiated thyroid cancer, *n* number of patients with available data evaluable

A total of 77.5% (165/213) of the aDTC population in the ERUDIT study was diagnosed with RR-DTC at any time during their metastatic life (Fig. 2) [15]. Demographic and clinical characteristics of the global study population at initial disease presentation are summarised in Supplementary Table 1. Briefly, 213 aDTC patients with median age of 63 years, 57% female, 54% were diagnosed as de novo aDTC, and 46% as recurrent/progressive eDTC.

ECOG performance status at RR-DTC diagnosis was recorded in 92/165 (55.8%), of whom 75/92 (81.5%) had ECOG 0–1 and 17/92 (18.5%) had ECOG 2–4; this missingness is addressed analytically by including a ‘missing’ category in multivariable models.

### Demographic, clinical, and treatment characteristics

RAI-refractoriness was diagnosed in 73.9% (85/115) of de novo aDTC patients and 81.6% (80/98) of the recurrent/progressive eDTC patients as such classified at their first aDTC presentation (Table 1). Almost eight percent (13/165) of total RR-DTC patients met at least one RAI refractoriness criterion at initial DTC diagnosis. Most patients, 68.5% (113/165), showed no RAI uptake in at least one lesion; although RAI refractoriness criteria were not mutually exclusive, and some patients met more than one. At RR-DTC diagnosis, 55.8% (92/165) of patients had ECOG status recorded. Of them, 81.5% (75/92) presented an ECOG status of 0–1 and 18.5% (17/92) an ECOG status of 2–4. Most commonly used technique to assess tumour extension in RR-DTC patients was computerised tomography (40.7%).

Table 2 summarises the therapeutic options administered to RR-DTC patients. WW strategy was used in 47.9% (79/165) of patients. Most frequent reason for selecting WW was the absence of clinically relevant progressive disease as found in 64.6% (51/79) of the patients. Median (Q1-Q3) WW duration was 31.3 (10.7–51.0) months. A total of 101 ST, distributed in several lines, were administered to 37.0% (61/165) of patients. The main MKIs used in this context included sorafenib 48.5% (49/101), lenvatinib 9.9% (10/101), and axitinib 9.9% (10/101). Most frequent reasons to discontinuation of ST were disease progression 56.4% (57/101), toxicity 11.9% (12/101), and death 5.9% (6/101). Generally, tumour progression was mainly monitored again by CT scans in 62.7% (74/118) of patients with available data.

**Table 2:** Therapeutic approaches received by the radioiodine-refractory population (*N* = 165)

Parameter	de novo aDTC (*N* = 85)	de novo aDTC (*N *= 85)	Global RAI-refractoriness DTC Population (*N* = 165)
Locoregional therapies	35 (41.1)	48 (60.0)	83 (50.3)
Re-surgery	7 (8.2)	18 (22.5)	25 (15.2)
Other locoregional therapies	28 (32.9)	30 (37.5)	58 (35.2)
Radiotherapies ^a^	27 (96.4)	27 (90.0)	54 (93.2)
Ablation therapies ^b^	1 (3.6)	3 (10.0)	4 (6.8)
Watchful waiting, patients n (%)	38 (44.7)	41 (51.3)	79 (47.9)
Most frequent reasons, patients *n* (%)			
No or minimally progressive advance disease	23 (60.5)	28 (68.3)	51 (64.6)
No symptoms	12 (31.6)	12 (29.3)	24 (30.4)
Duration, median (Q1-Q3), months	18.0 (7.4–42.4)	33.0 (12.6–64.9)	31.3 (10.7–51.0)
Lines of systemic therapy used, ^c^ patients *n* (%)	28 (32.9)	33 (41.3)	61 (37.0)
One	19 (22.4)	20 (25.0)	39 (23.6)
Two	4 (4.7)	7 (8.8)	11 (6.7)
Three	4 (4.7)	2 (2.5)	6 (3.6)
More	1 (1.2)	4 (5.0)	5 (3.0)
Most frequent reasons for ST discontinuation, *n* (%)	43	58	101
Disease progression	25 (58.1)	32 (55.2)	57 (56.4)
Toxicity	4 (9.3)	8 (13.8)	12 (11.9)
Death	3 (7.0)	3 (5.2)	6 (5.9)
Patient desire	1 (2.3)	2 (3.4)	3 (3.0)
Not available	10 (23.3)	13 (22.4)	23 (22.8)
Image test during follow-up, ^d^ patients *q* (%)	53 (62.4)	65 (81.3)	118 (71.5)
CT	34 (64.2)	40 (61.5)	74 (62.7)
^18^FDG-PET	12 (22.6)	12 (18.5)	24 (20.3)
Echography	3 (5.7)	7 (10.8)	10 (8.5)
WBS	1 (1.9)	2 (3.1)	3 (2.5)
Others	3 (5.7)	4 (6.2)	7 (5.9)

Criteria are not mutually exclusive allowing for a patient to be counted in more than one therapeutic approach. Though not expressly stated in the table, patients could have received > 3 systemic therapies; ^a^radiotherapies include external beam, modulated intensity, and stereotactic body radiotherapy, as well as radiofrequency, and thermal ablation; ^b^ablation therapies include thermal ablation with laser, ablation with radiofrequency, and alcohol injection; ^c^ reported systemic drugs included sorafenib (48.5%), lenvatinib (9.9.%), axitinib (9.9.%), sunitinib (8.9.%), pazopanib (6.9.%), and paclitaxel (3%) among others; ^d^ patients with at least one image test during follow-up

*aDTC* advanced differentiated thyroid cancer, *CT* computed tomography, *eDTC* early-stage of differentiated thyroid cancer, ^*18*^*FDG-PET* ^18^F-fluoro-2-deoxy-D-glucose positron emission tomography, *RAI* radioactive iodine (I-131), *ST* systemic therapy, WBS whole body scan

Sixty-four percent (106/165) of these cases were discussed in MDTs. Endocrinology department was the most frequently involved specialty monitoring 58.8% (97/165) of these patients, followed by medical oncology with 33.3% (55/165) and others (nuclear medicine, surgery, and internal medicine) with 4.8% (8/165) (Table 3).

**Table 3:** Medical specialities involved in the advanced disease/RAI-refractory management (*N* = 165)

Parameter	Global RAI-refractoriness DTC Population (*N* = 165)
Service responsible for patient monitoring, patient *n* (%)	97 (58.8)
Endocrinology	55 (33.3)
Oncology	4 (2.4)
Nuclear medicine	2 (1.2)
Surgery	1 (0.6)
Internal medicine	1 (0.6)
Others	5 (3.0)
Not available a	
Presence of multidisciplinary committee, patients *n* (%)	
No	53 (32.1)
Yes	106 (64.2)
Not available ^a^	6 (3.6)

^a^Records could not be retrieved from the electronic case report form

*DTC* differentiated thyroid cancer

*RAI* radioactive iodine (I-131)

### Time to event endpoints outcomes

The median (Q1-Q3) time from initial RAI treatment to RR-DTC diagnosis was 27.6 (9.5–50.6) months in 90% (150/165) patients with available data [20.2 (6.8–46.9) months in de novo aDTC cohort, and 35.7 (13.4–69.7) months in recurrent/progressive eDTC cohort]. At the end of the study period, only 61 (31%) RR-DTC patients had received ST; the median (Q1-Q3) time from RR-DTC diagnosis to start of ST (WW period), for those who did, was 8.9 (1.4–34.7) months [6.8 (0.6–30.1) months in de novo aDTC cohort, and 9.9 (2.7–35.0) months in recurrent/progressive eDTC cohort].

**Fig. 3:**
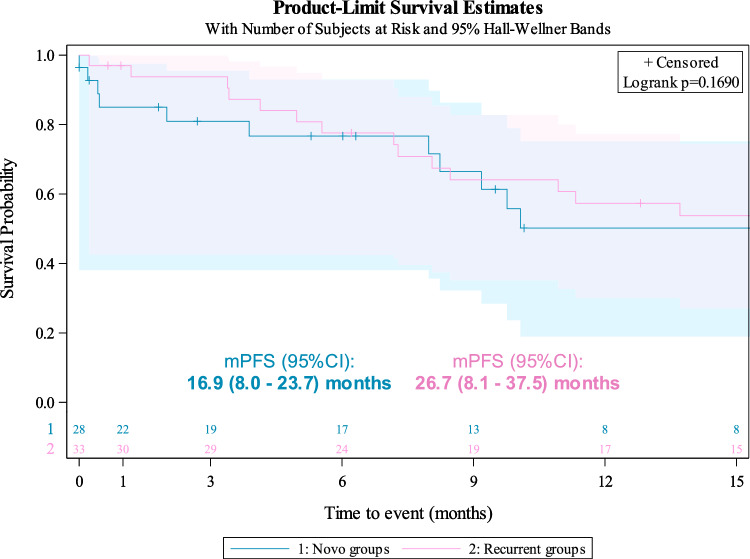
Progression-free survival (PFS) in RAI-refractory patients of first systemic therapy stratified by the time of advanced DTC (aDTC) diagnosis: de novo aDTC (blue) vs recurrent/progressive at the early-stage DTC (eDTC) (pink). No differences were found in the median PFS (log-rank test, *P* = 0.1690). mPFS (95% CI): median progression-free survival (95% CI) months

The median PFS (95%CI) of RR-DTC patients receiving first line ST was 16 (9.2–29.9) months. PFS for RR-DTC patients receiving a first line of ST did not statistically differ between de novo aDTC and recurrent/progressive eDTC cohorts [median PFS (95%CI): 16.9 (8.0–23.7) and 26.7 (8.1–37.5) months, respectively; log-rank test *P* = 0.1690] (Fig. 3).

**Fig. 4:**
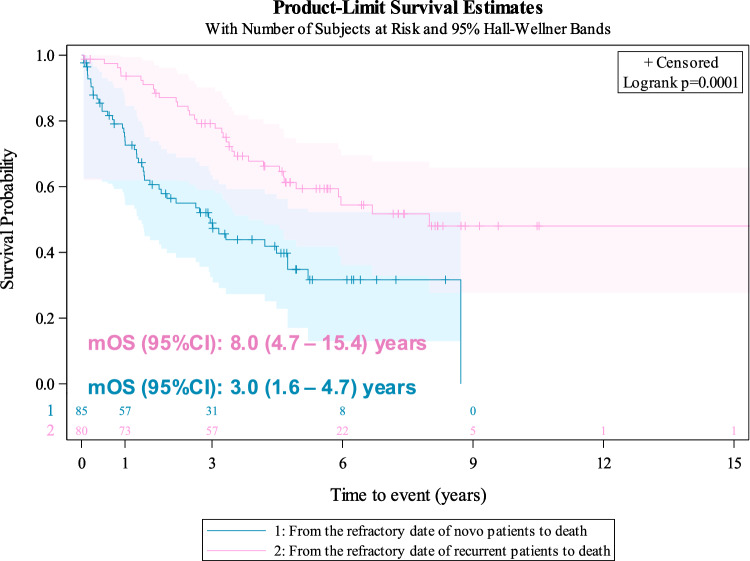
Kaplan–Meier overall survival (OS) curves measured from radioiodine-refractory DTC (RR-DTC) diagnosis to death in de novo aDTC (blue) vs recurrent/progressive early-stage DTC (eDTC) (pink) populations. The median OS of RR-DTC with de novo aDTC diagnosis was five years shorter than the OS of RR-DTC with recurrent/progressive eDTC diagnosis (log-rank test *P* < 0.0001)

Median OS (mOS) (95%CI) from RR-DTC diagnosis was 4.7 (3.4–8.0) years, significantly shorter in patients with de novo aDTC at initial DTC diagnosis than in recurrent/progressive eDTC patients [mOS (95%CI): 3.0 (1.6–4.7) vs 8.0 (4.7–15.4) years, respectively; log-rank test *P* < 0.0001] (Fig. 4).

**Fig. 5:**
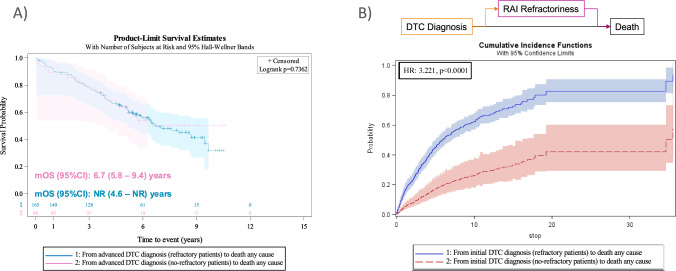
Overall survival (OS) curves. **A** Kaplan–Meier OS curves from the advanced differentiated thyroid cancer (DTC) diagnosis to death in the radioiodine-refractory DTC (RR-DTC) (*N* = 165, blue) population vs non-RR-DTC (*N* = 48, pink). No differences were found between the mOS of RR-DTC and the mOS of non-RR-DTC populations (log-rank *P* = 0.7362). **B** Uni-directional illness-death class of multi-state model, in which patients at their initial DTC diagnosis (excluding de novo RR-DTC) could progress via two alternative routes to death: 1) first becoming RAI-refractory and eventually dying, or 2) dying without becoming RAI-refractory first. Using a Markov multistep approximation, [20] the cumulative incidence of death was superior in RR-DTC (blue) than in non-RR-DTC patients (red) (hazard ratio = 3.22, *P* < 0.001). mOS (95% CI): median OS (95% CI) years

No statistically significant difference was found in the median OS from aDTC diagnosis between RR-DTC and non-RR-DTC patients [mOS (95%): 6.7 (5.8–9.4) and NE (4.6–NE) years, respectively; log-rank test *P* = 0.7362] (Fig. 5A). However, an additional Markov-based multistep model approximation showed a cumulative incidence curve from initial DTC diagnosis to death by any cause in RR-DTC above the curve of non-RR-DTC (HR: 3.22; *P* < 0.001) (Fig. 5B).

### Prognostic factors for OS

Univariate and multivariable Cox proportional hazards model showed that initial DTC diagnosis under 55 years [adjusted hazard ratio (aHR): 0.39, 95% CI: 0.21–0.75; Wald chi-square *P* = 0.0047] and WW duration superior to 30 months at RR-DTC diagnosis [aHR (95% CI): 0.29 (0.13–0.71); Wald chi-square *P* = 0.0059] were favourable independent prognostic factors for OS in the RR-DTC population. Conversely, de novo aDTC diagnosis [aHR (95% CI): 2.03 (1.17–3.52); Wald chi-square *P* = 0.0122] and ECOG status 2–4 at RR-DTC diagnosis [aHR (95% CI): 2.44 (1.23–4.81); Wald chi-square *P* = 0.0104] were associated with a higher risk of death (Table 4 and Supplementary Table 2).

**Table 4:** Multivariate analyses of overall survival from diagnosis of RAI-refractoriness to death or lost to follow-up (*N* = 165)

Covariates ^a^	N	Overall survival (Cox proportionalhazard model)	
		Adjusted hazard ratio (95%CI)	Wald chi-square *P*-value
General variables			
Age at diagnosis in years			
> = 55 (ref.)	113		
< 55	52	0.397 (0.209–0.754)	0.0047 ^b^
Variables of initial diagnosis and first treatment			
Diagnosis type			
Recurrent (ref.)	80		
de novo aDTC	85	2.026 (1.166–3.518)	0.0122 ^b^
Variables of advanced disease/RAI-refractory disease			
ECOG			
0–1 (ref.)	75		
2–4	17	2.435 (1.233–4.812)	0.0104 ^b^
Not available	73	1.344 (0.785–2.302)	0.2808
Watchful waiting duration, months			
< 30 (ref.)	38		
> = 30	41	0.298 (0.126–0.705)	0.0059 ^b^

^a^All covariates were entered into a Cox regression model; ^b^ statistically significant (*P* < 0.05)

(*ref*.) reference category, *aDTC* advanced differentiated thyroid cancer, *ECOG* eastern cooperative oncology group, *RAI* radioactive iodine (I-131), missing data were not imputed

## Discussion

Our results describe the clinicopathological features of a cohort of RR-DTC patients identified from a global study population of 213 patients with aDTC retrospectively followed for a median of 6.2 (4.5–9.0) years from the initial DTC diagnosis in the Iberian Peninsula [15]. Initial aDTC population included patients with first evidence of unresectable locally advanced and/or metastatic disease [presenting either de novo at initial DTC diagnosis or relapsed/progressed after first treatment(s)] during the inclusion period (August 2017 to August 2019).

To our knowledge, this is the first study to longitudinally characterize a multicenter, Iberian RR-DTC cohort arising from both de novo aDTC and recurrent/progressive eDTC. Beyond confirming known prognostic factors (age, ECOG), ERUDIT quantifies the specific contribution of regional management patterns like high MDT involvement, frequent WW attitude, or interesting findings like higher-than-expected RR-DTC incidence and a shorter interval to refractoriness among de novo aDTC patients with their corresponding survival impact. These data add geographic and health-system context to prior European and international cohorts and may inform region-specific optimization of surveillance and treatment timing.

We found that around 78% (165/213) of aDTC patients met one or more RAI refractoriness criteria at any time, with a median PFS of 16 months in patients receiving ST, and a median OS of 4.7 years from RR-DTC diagnosis. Initial DTC diagnosis in patients under 55 years and a WW duration superior to 30 months were in favour of prolonged survival, while de novo aDTC at initial DTC diagnosis and ECOG 2–4 at RR-DTC diagnosis negatively impacted OS.

According to our results, the incidence of RAI-refractoriness in aDTC patients found in the Iberian Peninsula is slightly superior to the 60–70% reported in the European guidelines for managing RR-DTC patients [3]. In our study, 48% of RR-DTC patients came from de novo aDTC at initial DTC diagnosis, while 51% came from recurrent/progressive eDTC. Around 92% of them were diagnosed as RR-DTC over the follow-up period with nearly 8% detected at first diagnosis. In general, non-RAI uptake in at least one lesion was the most frequent criterion (68.5%) in imaging tests. It is worth mentioning that 14.1% of de novo aDTC patients and 1.2% of recurrent/progressive eDTC met at least one RAI refractoriness criterion at the initial DTC diagnosis. Interestingly, in our study, aDTC patients with worst prognosis, took around 28 months (2.3 years) to become RAI-refractory, contrasting with the 3.3 years observed by Wassermann et al., 2016 [5]. This finding together with our higher RR-DTC incidence, suggests the ERUDIT aDTC sample was enriched with tumours carrying more aggressive features compared to those in other reports found in the general population [5].

For the first time, these data provide valuable information regarding the risk of RAI refractoriness in patients with de novo* aDTC* at initial DTC diagnosis and their remarkable short progression to RR-DTC (20.2 months) compared to those relapsing from eDTC (35.7 months). These results support the need for early detection of non-iodine avid metastatic lesions and premature disease progression. Frequency of thyroid US, CT, and 18F-fluoro-2-deoxy-D-glucose positron emission tomography (^18^FDG-PET) scans at initial DTC diagnosis as well as on periodical follow-up is still a matter of debate but their intensified use in these patients with markedly poor prognosis might improve detection of unusual premature disease progression prompting immediate therapeutic action.

At RR-DTC diagnosis, 82% of the patients presented with an ECOG score of 0–1 recorded, and 16% of those diagnosed with de novo aDTC having ECOG score of 2–4. According to our results, most patients followed an WW approach (47%) for a median time of 31 months until tumour progression was detected, as an acceptable option included in most clinical practice guidelines [13, 21]. Hence, patients were periodically followed using CT scans despite being asymptomatic in order to promptly detect early disease progression, initiate ST, and potentially optimise long-term clinical results.

Tumour progression was treated with locoregional therapies (50.3%), and/or ST approaches (37%). At the end of the study period (August 2017), 61 RR-DTC patients had initiated ST as rescue therapy after a median WW time of 8.9 months from the RR-DTC diagnosis, in line with the median time of 10.8 months described in RR-DTC patients that did not initiate MKI immediately in the RIFTOS MKI study [22]. Main treatments administered were sorafenib (48.5%) and lenvatinib (9.9%), the only approved drugs for the treatment of RR-DTC at the time of data collection.

Different medical specialties were involved in disease management of these patients mostly under the responsibility of MDT in over 64% of the cases after RR-DTC diagnosis. This finding is in line with current recommendations from clinical practice guidelines and expert consensus which suggest multidisciplinary evaluation of these tumours may have a positive impact in their prognosis [3, 12, 23]. Endocrinology was the primary responsible department, followed by medical oncology, nuclear medicine, surgery, and internal medicine.

PFS impact of sorafenib (10.8 months vs 5.8 months placebo) and lenvatinib (18.3 months vs 3.6 months placebo) in DECISION [10] and SELECT [8] phase III clinical trials, respectively, substantially changed the treatment landscape of advanced RR-DTC. Our study shows a median PFS for first-line ST of 16 months, very much in line with what other observational studies have previously described [22, 24]. Although PFS was numerically greater in patients with recurrent/progressive eDTC than those with de novo aDTC at initial diagnosis, the difference was not statistically significant. However, this numerical difference suggests again a worse prognosis for aDTC cases diagnosed de novo compared to those recurrent/progressive from prior treatment as we previously reported [15]. More research is needed to better understand the long-term impact of currently available therapeutic options in the survival expectancy of RR-DTC patients.

The median OS of RR-DTC patients in our cohort was 4.7 years, in line with previously published studies of similar characteristics [5, 6, 25]. However, it is something remarkable that in our study, the median OS of patients with de novo aDTC at initial diagnosis was significantly shorter than in patients with recurrent/progressive eDTC group (3 vs 8 years, respectively), confirming that early poor treatment-dependent prognostic factors (e.g., biochemical and/or structural response to initial RAI therapy) impact both the pace at which early disease progresses into advanced stages and the overall survival of aDTC patients once advanced disease is diagnosed [15].

Survival expectancies of RR-DTC patients and non-RR-DTC patients were surprisingly similar when measured from initial DTC diagnosis, contrasting with the expected shorter OS of RR-DTC patients as previously published [5]. This lack of a statistically significant difference in K-M OS, should however, be taken cautiously. We believe the overall worse clinical condition of the global study population, a short survival follow-up time with a limited number of deaths counted during the 6.2 (4.5–9.0) years and a complex case-mix likely attenuated the expected differences. Specifically, heterogeneity and evolution of RR-DTC definitions may lead to misclassification, while stage migration driven by sensitive imaging (e.g., FDG PET/CT) can shift case-mix across groups and narrow survival differences. Moreover, selection and investigator assessments biases inherent to multicenter retrospective designs, together with informative censoring, may distort OS estimates. Finally, non-cancer mortality in older/comorbid patients and unequal distribution of adverse molecular profiles (e.g., BRAF V600E/TERT promoter) can mask disease specific contrasts in all cause OS.

Although it is very difficult to account for these many factors, we strongly believe a longer survival follow-up would have very likely yielded the expected survival difference between RR-DTC patients and non-RR-DTC patients as it has been seen in other series.

In contrast, however, the cumulative probability of death from the initial DTC diagnosis to death by any cause in RR-DTC and non-RR-DTC patients determined using a unidirectional multistate model [20] explicitly accounting for the intermediate RR-DTC state, demonstrated a higher cumulative mortality for patients becoming RR-DTC, supporting clinical expectations while acknowledging model validity. The illness–death multistate approach was selected because our data feature an intermediate, clinically relevant state (RR-DTC) competing with alternative routes to death which are not adequately captured by single-event survival curves or separate competing-risk models. Multistate modelling provides pathway-aware cumulative mortality estimates (via Aalen–Johansen) from the time of initial diagnosis and allows transition-specific covariate effects; while it relies on a (possibly conditional) Markov assumption, this can be proved (e.g., time-since-entry as a covariate or formal tests) and, for state occupation probabilities, Aalen–Johansen estimates are known to be reasonably robust to mild departures from Markovity [26].

Despite these results come from a “post hoc” model approximation, we believe they support the importance of more closely assessing disease status in initial DTC patients with early signs of poor prognosis. This could lead to earlier detection of progressive disease and prompter therapeutic intervention that might overall impact in their survival expectancy.

Multivariable Cox model revealed that initial DTC diagnosis under 55 years and WW duration of more than 30 months were positive independent prognostic factors of longer OS in the RR-DTC population. Conversely, de novo aDTC at initial DTC diagnosis and ECOG status 2–4 at RR-DTC diagnosis were associated with a higher risk of death. In this study, patients with de novo aDTC at initial DTC diagnosis were more likely to have disease progression despite showing initial RAI uptake than those in the recurrent/progressive eDTC cohort (32.9% vs 22.5%, respectively; Table 1). Likewise, patients with de novo aDTC had shorter progression time to RR-DTC from first RAI than those relapsing to RR-DTC from the eDTC cohort (20.2 months vs 35.7 months, respectively). These results are in line with shorter median OS found by Wasserman et al., [5] and Durante et al., [6] in patients whose disease progressed despite RAI uptake and after short progression times to RR-DTC from first diagnosis. This exemplifies the heterogeneous nature of these tumours, where despite an initial response to RAI, theoretically, some ‘non-responding RAI clones’ may lead to disease progression. Interestingly, the negative impact of RAI refractoriness at the initial DTC diagnosis (7.9%) on OS was statistically significant only in the univariate analysis but not in the adjusted multivariable Cox model. We believe this could be possibly explained by the limited sample size of this group in our study population. In this multivariable analysis, however, we did not include the time from initial DTC diagnosis to RR-DTC diagnosis which, similar to Wasserman et al., [5], we expect would have resulted an independent significant prognostic factor. Based on these findings, we agree with other authors that the timely diagnosis of RAI refractoriness will assist in implementing therapeutic measures that could significantly change the life expectancy and potentially impact the quality of life of these patients [5, 6].

On the other hand, based on our findings, the association suggested between a longer WW (≥ 30 months) and improved OS, although provocative, is potentially vulnerable to selection, lead-time, and immortal-time biases. Patients suitable for prolonged WW are likely have tumours with less aggressive biology, a more favourable disease burden/distribution, and better performance status overall. Therefore, we avoid making a strict causal interpretation while rather understand WW as a management pattern associated with outcomes that warrants prospective evaluation with bias-resistant designs (e.g., landmark analyses, time-dependent covariates, or randomized strategies where feasible).

This study has several limitations. Its retrospective, multicentre design and limited sample size may have introduced selection bias, affected group distribution, and reduced the statistical power of uni- and multivariable analyses. Centre-level heterogeneity in work-up, follow-up intensity, and therapeutic approaches could also contribute to reduce overall data uniformity. Missing data, particularly ECOG at RR-DTC diagnosis, were not imputed to avoid unverifiable assumptions; instead, a “missing” category was used in multivariable Cox model, which may yield conservative estimates if missingness correlates with worse baseline status. Although we restricted covariates to mitigate overfitting, the number of events constrains precision and widens confidence intervals. In addition, the use of physician-reported responses (rather than central blinded review) and the lack of standardized criteria for initiating or extending watchful waiting (WW) across centres may have introduced information and selection biases. Some potentially relevant variables (e.g., detailed molecular profiling or granular disease burden/distribution metrics) were not systematically captured, limiting adjustment for residual confounding.

Period effects (2007–2012 diagnoses) and regional practice patterns further limit generalizability, as the cohort predates the widespread use of later-line agents (e.g., cabozantinib) and contemporary systemic combinations. Consequently, absolute outcome estimates may underrepresent the benefits achievable with newer therapies and current supportive care. Our illness–death multistate analysis was prespecified as exploratory and relies on Markov assumptions (e.g., memoryless transitions) that may not fully hold in heterogeneous real-world pathways; results should therefore be interpreted as complementary to Kaplan–Meier rather than definitive. Taken together, these factors, along with potential lead-time and immortal-time biases, preclude causal inference regarding WW. The associations reported should be considered hypothesis-generating and warrant confirmation using bias-resistant designs (e.g., time-dependent covariates, landmark analyses, or prospective studies).

On the other hand, this study longitudinally follows for the first time relevant clinical data of a sizeable cohort of European RR-DTC patients first diagnosed as aDTC de novo or recurrent/progressive from initial eDTC that provides valuable information on the clinicopathological characteristics, therapeutic management, and prognostic information useful in everyday practice decision-making.

Similar future epidemiological investigations should include currently available STs for the treatment of RR-DTC to better understand the individual impact they have had in this difficult-to-treat population and how they have helped improving clinical practice in the Iberian Peninsula.

## Conclusion

The study reveals that 78% with aDTC diagnosis in the Iberian Peninsula became RR-DTC around two years after their first RAI treatment. Patients receiving ST showed a median PFS of 16 months. Median OS expectancy was 4.7 years. De novo aDTC at initial DTC diagnosis and ECOG 2–4 at RR-DTC diagnosis negatively affected OS. WW also appeared associated with longer OS, but this may reflect selection and lead/immortal-time biases, underscoring the need for prospective validation.

These findings support the importance of more closely assessing disease status in RR-DTC patients with early signs of poor prognosis. This could lead to earlier detection of progressive disease and prompter therapeutic intervention overall impacting their survival expectancy and quality of life.

## Supplementary Information

Below is the link to the electronic supplementary material.

Supplementary file1 (DOCX 25 KB)

## Data Availability

Not applicable.

## Abbreviations

**aDTC: **: Advanced differentiated thyroid cancer
**aHR: **: Adjusted hazard ratio
**CT: **: Computed tomography
**DTC: **: Differentiated thyroid cancer
**ECOG: **: Eastern cooperative oncology group
**eDTC: **: Early Stage differentiated thyroid cancer
**K-M: **: Kaplan–Meier
**MDTs: **: Multidisciplinary teams
**MKIs: **: Multi-tyrosine kinase inhibitors
**OS: **: Overall survival
^**18**^**FDG****-PET****: **: ^18^F-Fluoro-2-deoxy-D-glucose positron emission tomography
**PFS: **: Progression-free survival
**RAI: **: I-131 Radioiodine
**RR-DTC: **: Radioiodine-refractory differentiated thyroid cancer
**RR: **: RAI-refractory
**ST: **: Systemic therapy
**WBS: **: Whole body scan
**Wc-s: **: Wald chi-square
**WW: **: Watchful waiting
**95****%CI: **: 95% Confidence interval
