# Supplementary material for: Clinical and treatment patterns of advanced and radioiodine-refractory differentiated thyroid cancer. ERUDIT study
Source: Clin Transl Oncol. 2025 Nov 19;28(5):1839–52. doi: 10.1007/s12094-025-04122-6 (PMC13099722; doi:10.1007/s12094-025-04122-6)
Supplement: Supplementary file 1 — Supplementary file1 (DOCX 25 KB) [file 12094_2025_4122_MOESM1_ESM.docx]

Supplementary Table 1. Demographic and clinical characteristics of the study population at initial disease presentation (N=213)^15^

| **Parameter** | ***de novo***  **aDTC** | **Recurrent / Progressive**  **eDTC** | **Global Study**  **Population** |
| --- | --- | --- | --- |
| Patients, n (%) | 115 (54.0) | 98 (46.0) | 213 (100) |
| Age at initial diagnosis, median (Q1-Q3), years. | 67.0 (57.0 - 73.0) | 56.5 (45.0 - 67.0) | 63.0 (51.0 - 71.0) |
| Gender, patients n (%) |  |  |  |
| Female | 65 (56.5) | 61 (62.2) | 126 (59.2) |
| Male | 50 (43.5) | 37 (37.8) | 87 (40.8) |
| Comorbidities, patients n (%) |  |  |  |
| ≥ 1 comorbidity | 50 (43.5) | 41 (41.8) | 91 (42.7) |
| Cardiovascular | 33 (28.7) | 28 (28.6) | 61 (28.6) |
| Metabolic | 26 (22.6) | 14 (14.3) | 40 (18.8) |
| Other clinically relevant | 13 (11.3) | 10 (10.2) | 23 (10.8) |
| Initial diagnosis of DTC and method, patients n (%) |  |  |  |
| Incidental post- surgery | 15 (13.0) | 30 (30.6) | 45 (21.1) |
| No incidental | 100 (87.0) | 68 (69.4) | 168 (78.9) |
| Echography | 54 (54.0) | 47 (69.1) | 101 (60.1) |
| Others | 45 (45.0) | 20 (29.4) | 65 (38.7) |
| Not available | 1 (1.0) | 1 (1.5) | 2 (1.2) |
| Fine needle aspiration result, patients n (%) | 71 (61.7) | 58 (59.2) | 129 (60.6) |
| Malignant | 60 (84.5) | 40 (69.0) | 100 (77.5) |
| Indeterminate | 4 (5.6) | 13 (22.4) | 17 (13.2) |
| Benign | 5 (7.0) | 1 (1.7) | 6 (4.7) |
| Nondiagnostic | 2 (2.8) | 4 (6.9) | 6 (4.7) |
| Histological result, patients n (%) |  |  |  |
| Papillary thyroid carcinoma | 67 (60.4) | 58 (59.2) | 125 (59.8) |
| Follicular thyroid carcinoma | 26 (23.4) | 13 (13.3) | 39 (18.7) |
| Oncocytic carcinoma (Hürthle cell carcinoma), ^a^ | 5 (4.5) | 16 (16.3) | 21 (10.0) |
| Poorly differentiated | 5 (4.5) | 3 (3.1) | 8 (3.8) |
| Mixed carcinoma (papillary and follicular) | 4 (3.6) | 3 (3.1) | 7 (3.3) |
| Others | 4 (3.6) | 5 (5.1) | 9 (4.3) |
| Biochemistry at diagnostic, ^b^ patients, n (%) | 42 (36.5) | 32 (32.7) | 74 (34.7) |
| Diagnosis images, patients n (%) | 93 (51.7) | 87 (48.3) | 180 (100) |
| Measurable disease, n (%) | 58 (62.4) | 48 (55.2) | 106 (58.9) |
| Tumour size, median (mm) (Q1-Q3) | 40.0 (26.0 - 60.0) | 38.5 (25.0 - 50.5) | 40.0 (25.0 - 57.0) |
| Lobes involved, n (%) |  |  |  |
| Left or right | 60 (64.5) | 59 (67.8) | 119 (66.1) |
| Both | 27 (29.0) | 24 (27.6) | 51 (28.3) |
| Not available  Tumour invasion,^c^ n (%) | 6 (6.5) | 4 (4.6) | 10 (5.6) |
| Present | 34 (36.6) | 14 (16.1) | 48 (26.7) |
| Absent | 12 (12.9) | 22 (25.3) | 34 (18.9) |
| Unknown | 39 (41.9) | 39 (44.8) | 78 (43.3) |
| Pathological lymph nodes | 24 (25.8) | 13 (14.9) | 37 (20.6) |
| Metastases by site, n (%) Lung | 66 (71.0) | ^d^ | 66 (71.0) |
| Liver | 5 (5.4) | ^d^ | 5 (5.4) |
| Bone | 33 (35.5) | ^d^ | 33 (35.5) |
| Others | 3 (3.2) | ^d^ | 3 (3.2) |

aWHO Classification of Thyroid Neoplasms update^16^; ^b^Refers patients with thyroglobulin (Tg): Tg levels and/or anti-Tg positive in serum; ^c^Tumour invasion refers to disease invading either adjacent tissues and structures and/or vascular spaces. Patients could be part of more than one tumour invasion category making statistical testing not feasible; drecurrent / progressive eDTC patients, by definition, had no metastases at diagnosis. aDTC, advanced differentiated thyroid cancer; eDTC, early-stage of differentiated thyroid cancer.

Supplementary Table 2. Kaplan-Meier univariate analysis of overall survival from diagnosis of RAI-refractoriness to death or lost to follow-up (N=165)

| **Parameters** | **N** | Overall Survival (Kaplan-Meier) | | |
| --- | --- | --- | --- | --- |
|  |  | **Median (95%CI)** | **Hazard ratio (95%CI)** | **Log-rank P-value** |
| **General variables** |  |  |  |  |
| Age at diagnosis in years |  |  |  |  |
| >=55 (Ref.) | 113 | 40.7 (31.8 - 55.7) |  |  |
| <55 | 52 | 184.9 (NE - NE) | 0.312 (0.167 - 0.540) | <.0001 ^d^ |
| >=45 (Ref.) | 138 | 50.6 (36.9 - 62.5) |  |  |
| <45 | 27 | 184.9 (NE - NE) | 0.259 (0.091 - 0.580) | 0.0017 ^d^ |
| **Variables of initial diagnosis and first treatment** |  | | | |
| Diagnosis type |  |  |  |  |
| Recurrent (Ref.) | 80 | 95.9 (55.9 - 184.9) |  |  |
| Novo | 85 | 35.5 (18.8 - 56.8) | 2.363 (1.511 - 3.743) | 0.0001 ^d^ |
| Surgical outcome |  |  |  |  |
| R2 (Ref.) | 20 | 26.1 (5.7 - 39.8) |  |  |
| R0/R1 | 129 | 62.5 (46.1 - NE) | 0.407 (0.236 - 0.748) | 0.0034 ^d^ |
| Others ^a^ | 11 | 104.6 (10.9 - 184.9) | 0.293 (0.094 - 0.770) |  |
| Ablative RAI +/- local therapies |  |  |  |  |
| Yes (Ref.) | 150 | 59.2 (46.1 - 184.9) |  |  |
| No | 15 | 12.0 (1.6 - 55.7) | 2.624 (1.350 - 4.666) | 0.0014 ^d^ |
| RAI Refractoriness at initial diagnosis |  |  |  |  |
| No (Ref.) | 152 | 59.2 (46.1 - 184.9) |  |  |
| Yes | 13 | 8.5 (1.6 - 36.2) | 3.007 (1.503 - 5.453) | 0.0004 ^d^ |
| RAI Accumulated dose |  |  |  |  |
| >=600 mCi (Ref.) | 28 | 62.5 (23.5 - 184.9) |  |  |
| <600 mCi | 121 | 59.2 (46.1 - NE) | 1.090 (0.583 - 2.269) | 0.0052 ^d^ |
| No RAI treatment ^b^ | 15 | 12.0 (1.6 - 55.7) | 2.857 (1.231- 6.776) |  |
| **Variables of 1st relapse** |  |  |  |  |
| Type of relapse |  |  |  |  |
| Structural (Ref.) | 54 | 55.7 (39.1 - 184.9) |  |  |
| Both | 53 | 71.5 (50.2 - NE) | 0.850 (0.477 - 1.499) | 0.0258 ^d^ |
| Not relapse | 58 | 36.2 (22.0 - 56.9) | 1.684 (1.004 - 2.855) |  |
| Variables of advanced disease / RAI-refractroy disease ^c^ |  |  |  |  |
| RAI refractoriness criteria 1 |  |  |  |  |
| No (Ref.) | 128 | 53.8 (35.5 - 104.6) |  |  |
| Yes | 37 | 70.8 (50.2 - NE) | 0.761 (0.431 - 1.272) | 0.3175 |
| RAI refractoriness criteria 2+3+4 |  |  |  |  |
| No (Ref.) | 70 | 55.7 (39.8 - 71.5) |  |  |
| Yes | 95 | 56.9 (36.2 - 184.9) | 0.792 (0.511 - 1.229) | 0.2956 |
| RAI refractoriness criteria 5 |  |  |  |  |
| No (Ref.) | 116 | 53.8 (36.2 - 70.8) |  |  |
| Yes | 49 | 95.9 (46.1 - 184.9) | 0.636 (0.374 - 1.034) | 0.0763 |
| RAI refractoriness criteria 6 |  |  |  |  |
| No (Ref.) | 119 | 50.6 (37.9 - 70.8) |  |  |
| Yes | 46 | 184.9 (38.7 - 184.9) | 0.502 (0.265 - 0.877) | 0.0202 ^d^ |
| RAI refractoriness criteria 7+8 |  |  |  |  |
| No (Ref.) | 135 | 59.2 (39.9 - 95.9) |  |  |
| Yes | 30 | 41.5 (11.6 - NE) | 1.304 (0.749 - 2.156) | 0.3214 |
| ECOG |  |  |  |  |
| 0-1 (Ref.) | 75 | 104.6 (55.9 - NE) |  |  |
| 2-4 | 17 | 9.0 (2.6 - 35.1) | 5.695 (3.045 - 10.331) | <0.0001^d^ |
| Not available | 73 | 42.1 (29.8 - 184.9) | 1.624 (0.992 - 2.678) |  |
| Watchful waiting |  |  |  |  |
| Yes (Ref.) | 79 | 104.6 (70.8 - NE) |  |  |
| No | 83 | 29.4 (18.8 - 53.8) | 2.861 (1.801 - 4.656) | <0.0001^d^ |
| Watchful waiting duration (months) |  |  |  |  |
| <30 (Ref.) | 38 | 41.5 (31.5 - NE) |  |  |
| >=30 | 41 | NE (80.2 - NE) | 0.199 (0.080 - 0.457) | <0.0001^d^ |
| Radiotherapy for advanced/RAI-refractory disease |  |  |  |  |
| No (Ref.) | 111 | NE (55.7 - NE) |  |  |
| Yes | 54 | 36.9 (29.4 - 50.2) | 2.171 (1.399 - 3.368) | 0.0004 ^d^ |
| Reason for systemic therapies discontinuation |  |  |  |  |
| Disease progression (Ref.) | 35 | 39.1 (20.1 - 56.8) |  |  |
| Toxicity or patient desire | 7 | 54.6 (3.1 - NE) | 0.768 (0.225 - 2.001) | 0.0023 ^d^ |
| Death | 3 | 46.1 (17.4 - 95.9) | 1.083 (0.257 - 3.102) |  |
| Non-discontinuation | 16 | NE (NE - NE) | 0.000 (NE - 0.137) |  |

a Resection of other lesions such as adenopathy or metastasis; ^b^ patients not receiving RAI because of it not being clinically indicated or having tumours with negative RAI scans; ^c^ RAI refractoriness criteria^18^ (1, there was no uptake of RAI in the initial diagnosis of distant metastases or locoregional recurrence; 2, progressive loss of RAI uptake after several RAI therapy sessions; 3, evidence of different outbreak of metastases, some of them with RAI absorption and others without RAI absorption in the body; 4, tumour progression within the first year after treatment with adequate RAI even with substantial iodine uptake; 5, significant uptake in 18F-fluoro-2-deoxy-D-glucose positron emission tomography; 6, total cumulative RAI doses of more than 600 mCi; 7, unresectable primary tumours of DTC; and 8, aggressive DTC histologies (such as non-differentiated oncocytic carcinoma or insular carcinomas); ^d^ statistically significant (p<0.05). (ref.): reference category; CI: confidence interval; NE: non-estimable; R0: microscopic complete resection; R1: macroscopic resection with microscopic residual tumour; R2: gross macroscopic residual tumour; RAI, radioactive Iodine (I-131); missing data were not imputed.
